# Measuring the accuracy of cardiac output using POCUS: the introduction of artificial intelligence into routine care

**DOI:** 10.1186/s13089-022-00301-6

**Published:** 2022-12-14

**Authors:** Faisal Shaikh, Jon-Emile Kenny, Omar Awan, Daniela Markovic, Oren Friedman, Tao He, Sidharth Singh, Peter Yan, Nida Qadir, Igor Barjaktarevic

**Affiliations:** 1 Division of Interventional Pulmonology, Beth Israel Medical Center and Massachusetts General Hospital, Boston, MA USA; 2 Health Sciences North Research Institute, Sudbury, ON Canada; 3 Flosonics Medical, Sudbury, ON Canada; 4 Division of Pulmonary and Critical Care, Washington DC VA Medical Center, Washington, DC USA; 5 Department of Medicine Statistics, David Geffen School of Medicine at University of California, Los Angeles, CA USA; 6 Division of Cardiothoracic Surgery, Cedars Sinai Medical Center, Los Angeles, CA USA; 7 Division of Pulmonary and Critical Care, David Geffen School of Medicine at University of California Los Angeles, Los Angeles, CA USA; 8 Division of Pulmonary and Critical Care Medicine, David Geffen School of Medicine, University of California Los Angeles, 10833 Le Conte Avenue, CHS Building, 43118, Los Angeles, CA 90095 USA

**Keywords:** Velocity time integral, VTI, Point-of-care ultrasound, POCUS, Hemodynamic monitoring, Cardiac output, Artificial intelligence

## Abstract

**Background:**

Shock management requires quick and reliable means to monitor the hemodynamic effects of fluid resuscitation. Point-of-care ultrasound (POCUS) is a relatively quick and non-invasive imaging technique capable of capturing cardiac output (CO) variations in acute settings. However, POCUS is plagued by variable operator skill and interpretation. Artificial intelligence may assist healthcare professionals obtain more objective and precise measurements during ultrasound imaging, thus increasing usability among users with varying experience. In this feasibility study, we compared the performance of novice POCUS users in measuring CO with manual techniques to a novel automation-assisted technique that provides real-time feedback to correct image acquisition for optimal aortic outflow velocity measurement.

**Methods:**

28 junior critical care trainees with limited experience in POCUS performed manual and automation-assisted CO measurements on a single healthy volunteer. CO measurements were obtained using left ventricular outflow tract (LVOT) velocity time integral (VTI) and LVOT diameter. Measurements obtained by study subjects were compared to those taken by board-certified echocardiographers. Comparative analyses were performed using Spearman’s rank correlation and Bland–Altman matched-pairs analysis.

**Results:**

Adequate image acquisition was 100% feasible. The correlation between manual and automated VTI values was not significant (*p* = 0.11) and means from both groups underestimated the mean values obtained by board-certified echocardiographers. Automated measurements of VTI in the trainee cohort were found to have more reproducibility, narrower measurement range (6.2 vs. 10.3 cm), and reduced standard deviation (1.98 vs. 2.33 cm) compared to manual measurements. The coefficient of variation across raters was 11.5%, 13.6% and 15.4% for board-certified echocardiographers, automated, and manual VTI tracing, respectively.

**Conclusions:**

Our study demonstrates that novel automation-assisted VTI is feasible and can decrease variability while increasing precision in CO measurement. These results support the use of artificial intelligence-augmented image acquisition in routine critical care ultrasound and may have a role for evaluating the response of CO to hemodynamic interventions. Further investigations into artificial intelligence-assisted ultrasound systems in clinical settings are warranted.

## Background

While multiple methods for measuring cardiac output (CO) exist [1], methods like point-of-care ultrasound (POCUS) and transthoracic echocardiography are increasingly popular given their non-invasive nature, cost-effectiveness, and availability in most healthcare settings [2–4]. Specifically, POCUS allows for repeated assessment of CO during therapeutic maneuvers in shock management [5]. Ultrasonographic assessment of CO is calculated from velocity time integral (VTI) via the left ventricular outflow tract (LVOT). LVOT VTI is often preferred for measurement because it is easily measurable, has good inter/intra-observer rater reliability, and correlates strongly with PAC (pulmonary artery catheter) thermodilution [6]. As such, using VTI for CO measurements is a reasonable surrogate for invasive estimates for CO as determined by ESICM (European Society of Intensive Care Medicine) consensus guidelines [7].

In practice, however, VTI has been criticized for poor accuracy and reproducibility [6, 8]. However, automation and artificial intelligence (AI) may mitigate some of the VTI’s shortcomings in POCUS. AI now allows for real-time user feedback during image acquisition, which may reduce human error and narrow discrepancies in training. To evaluate accuracy, reproducibility, and feasibility of AI-assisted ultrasound, we conducted a study testing the ability of novice ultrasound (US) trainees to measure CO using automation-assisted VTI (VTI_auto_).

## Methods

### Participants

This prospective study was conducted at a regional POCUS training conference with critical care trainees from five academic centers in Southern California [9]. We recruited first-year fellows in critical care fellowships with limited experience in echocardiography. All VTI studies were conducted following an 8-h didactic session and a preliminary hands-on training session on obtaining basic echocardiographic windows. All study participants, including both trainees and study subject, provided informed consent. The Institutional Review Board at University of California Los Angeles has approved this study (UCLA IRB# 20-000690).

### Measurements

Multiple consecutive US assessments of CO were performed on a single healthy volunteer placed in the lateral decubitus position. Over a period of 3 h, 28 first-year critical care trainees performed manual and automated LVOT VTI measurements. For both manual and automated measurements, LVOT diameter (D_LVOT_) was calculated in the parasternal long axis (PLAX) position. Aortic outflow velocities were obtained by placing the pulsed-wave Doppler tracer in the LVOT immediately proximal to the aortic valve cusps in the apical 5-chamber (AP5c) view. Only the largest of five consecutive beats were selected to account for respiratory variation. While no direct assistance was given to trainees, image acceptability was verified by two senior fellows in critical care medicine with 3 years of formal training in basic and advanced ultrasonography.

**Fig. 1:**
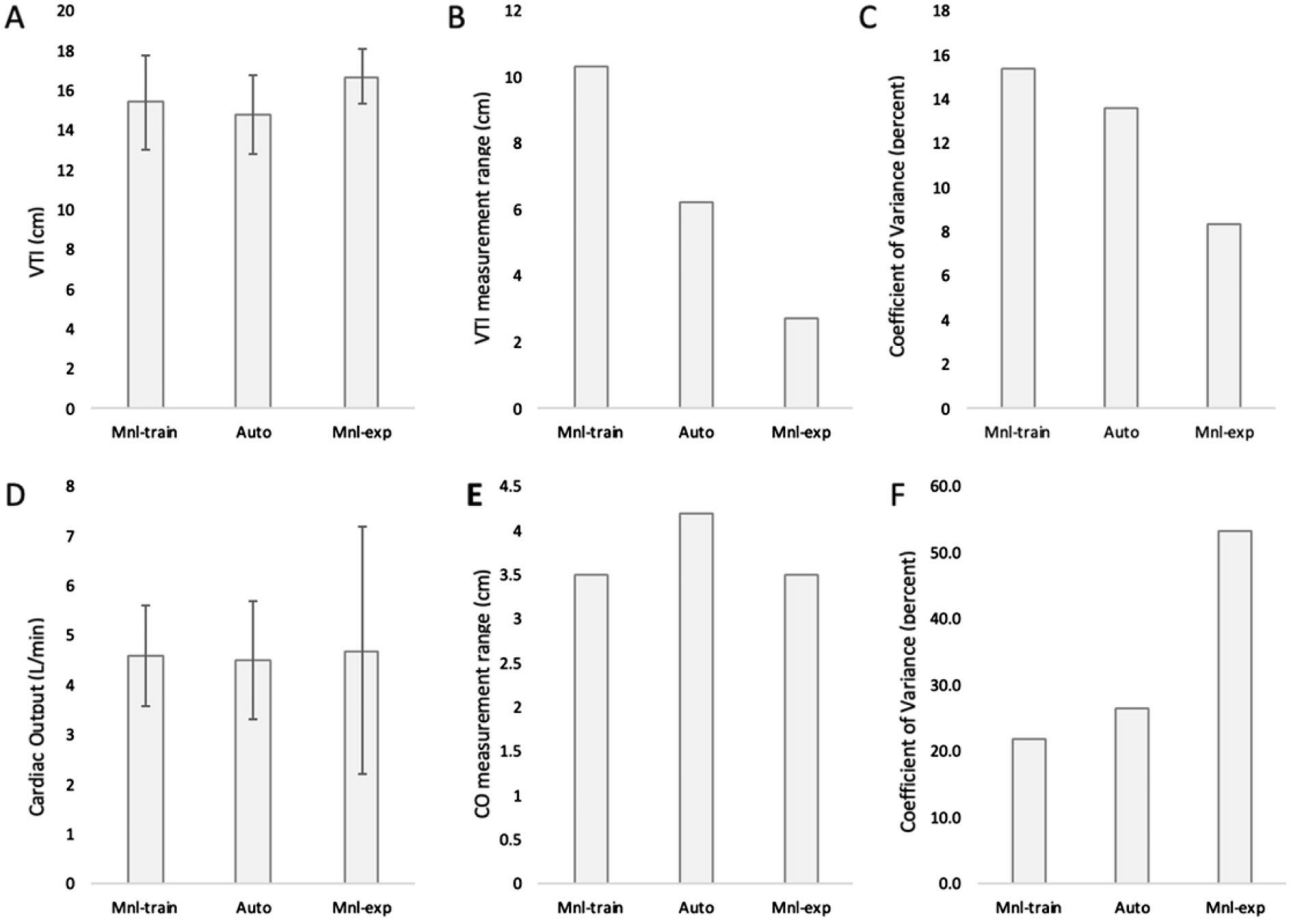
Comparison between manually obtained measures by trainees (Mnl-train), automated measurements (Auto), and manual measurements of certified ultrasonographers (Mnl-exp). **A** Mean velocity time integral (VTI), **B** range of obtained VTI measurements, **C** coefficient of variance of VTI measures, **D** mean cardiac output (CO), **E** range of obtained CO measurements, **F** coefficient of variance of CO measure

Manual VTI measurements by trainees (VTI_mnl-train_) were calculated by tracing the spectral envelope of the single-best beat with the largest, most clearly visible Doppler profile (determined with the assistance of senior fellows). CO was then calculated using the modified Bernoulli’s equation (CO = VTI × (D_LVOT_/2)^2^ × *π* × HR). VTI_auto_ was calculated using the ultrasound manufacturer’s propriety software (*Venue Go, GE Healthcare, Waukesha, WI*). This software included real-time feedback to correct image acquisition for optimal aortic outflow velocity measurement, along with fully automated spectral envelop tracing. The measurements of 5 sequential beats were then used as part of the automated algorithm calculation for VTI_auto_ and automated CO (CO_auto_). The averages of three separate, manual VTI measurements obtained by two board-certified echocardiographers (VTI_mnl-exp_) were used as reference for VTI and CO. These measurements were performed at the beginning and end of the study on the same volunteer to capture any physiologic changes in VTI that may have occurred over the course of the study (approximately 3 h) (Fig. 1).

### Statistical analysis

Feasibility of image capture was defined as the percent of trainees able to acquire adequate PLAX and AP5c views for D_LVOT_ and LVOT-VTI measurements. We evaluated the inter-rater reproducibility of both manual and automated VTI tracing and assessed the range of measurements, the standard deviation, and coefficient of variation between these two measurements. We also assessed the agreement between automated and manual measurements using Bland–Altman matched-pairs analysis. To determine accuracy, we used D_LVOT_ and VTI obtained by advanced echocardiographers as reference measurements for cardiac measurements obtained by novice US users.

## Results

### Baseline characteristics of novice echocardiographers

Mean self-reported experience in POCUS was 2.6 ± 2 years. 12 of 28 participants stated they had received prior ultrasound training; however, none had previous training in VTI or obtaining CO estimates through US (Table 1). In terms of feasibility, all 28 participants (100%) were able to obtain ultrasonographic views adequate for capturing required metrics of VTI and CO after hands-on training.

**Table 1:** Operators’ characteristics and study measurements

Operators’ characteristics	
Number of trainees (*N*)	28
Number of expert ultrasonographers (*N*)	2
Years of experience, mean ± SD	2.6 ± 2.0
Post-graduate year in training, mean ± SD	4.6 ± 1.4
Previous general US training (%)	44.4

Study measurements	
Heart rate, mean ± SD	65.8 ± 5.0
VTI-auto (cm), mean ± SD	14.8 ± 2.0
VTI-mnl-trainee (cm), mean ± SD	15.2 ± 2.4
VTI-mnl exp (cm), mean ± SD	16.7 ± 1.9
LVOT-mnl-trainee (cm), mean ± SD	2.4 ± 0.2
LVOT-mnl-exp (cm), mean ± SD	2.5 ± 0.4
CO-auto (L/min), mean ± SD	4.5 ± 1.2
CO-mnl-trainee (L/min), mean ± SD	4.6 ± 1.0
CO-mnl-exp (L/min), mean ± SD	5.7 ± 2.5

### Precision and reproducibility of VTI measurements

**Fig. 2:**
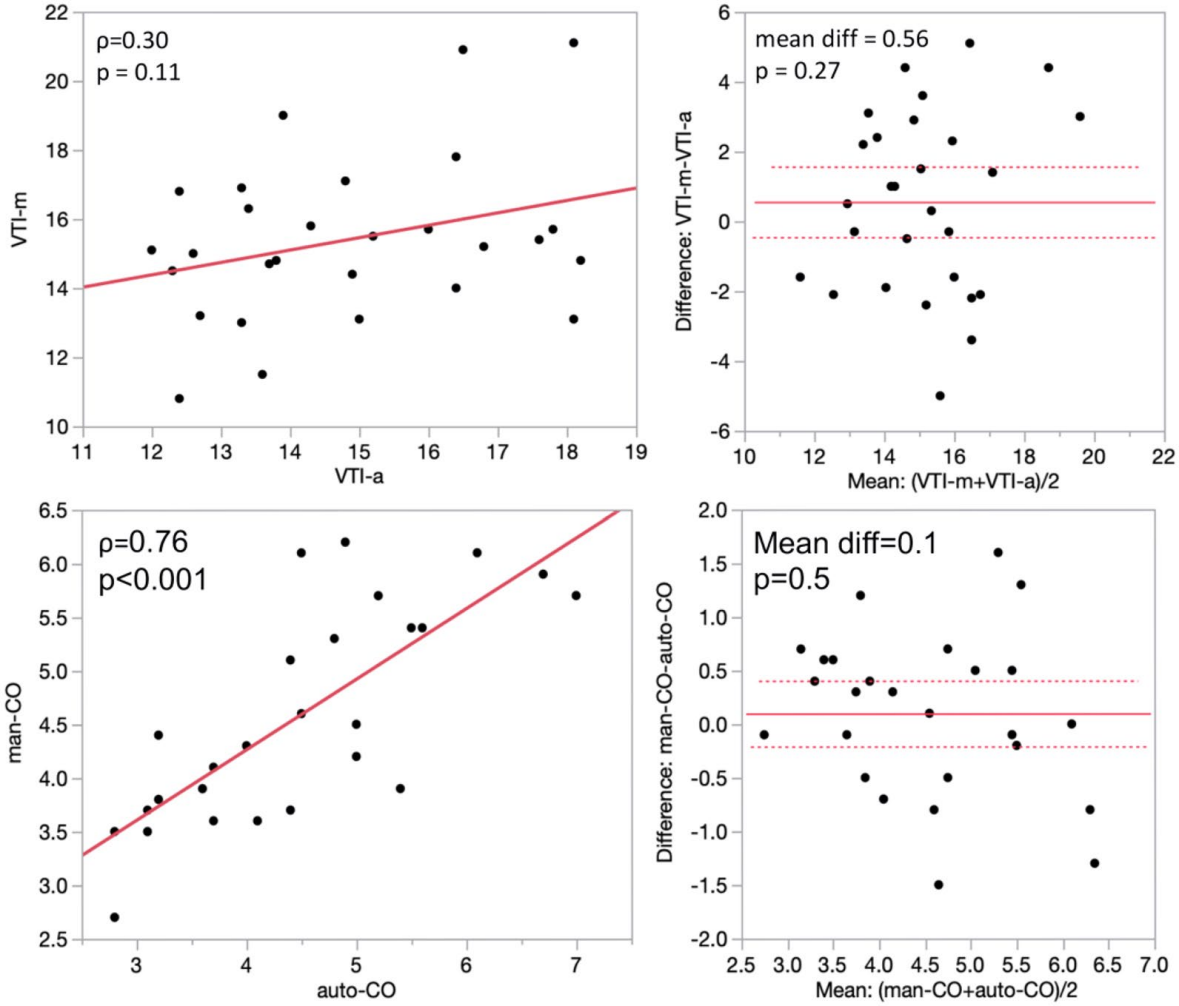
Comparative analysis of trainee-obtained manual measurements (VTI_mnl-train_ and CO_mnl-train_) and automated measurements (VTI_auto_ and CO_auto_). **A** Correlation between VTI_mnl-train_ and VTI_auto_, **B** Bland–Altman agreement analysis between VTI_mnl-train_ and VTI_auto_, **C** correlation between CO_mnl-train_ and CO_auto_, **D** Bland–Altman agreement analysis between CO_mnl-train_ and CO_auto_

Measurement results are presented in Table 1. The average VTI_auto_ obtained by trainees was 14.8 ± 2.0 cm, while the average VTI_mnl-train_ was 15.4 ± 2.3 cm. The correlation between VTI_auto_ and VTI_mnl-train_ was not significant (*ρ* = 0.30, *p* = 0.11) (Fig. 2a). Using Bland–Altman matched-pairs analysis, we determined that the mean paired difference between VTI_mnl-train_ and VTI_auto_ was not significantly different from zero (mean difference = 0.56, *p* = 0.27), indicating that there was no systematic bias between the two methods (Fig. 2b). The 95% limits of agreement were between − 4.6 and + 5.6. VTI_auto_ was more reproducible across raters than VTI_mnl-train_, with a narrower measurement range (6.2 cm vs. 10.3 cm), and smaller standard deviation (1.98 cm vs. 2.33 cm). The coefficient of variation across raters (i.e., the standard deviation relative to the mean) was smaller for VTI_auto_ compared to VTI_mnl-train_ (13.6% vs. 15.4%). The coefficient of variation was lowest (11.5%) for our control group of advanced echocardiographers.

### Accuracy of VTI measurements

Both mean VTI_auto_ (14.8 ± 2.0 cm) and mean VTI_mnl-train_ (15.4 ± 2.4 cm) underestimated the mean VTI obtained by the two advanced echocardiographers (16.7 ± 1.4 cm). Considering VTI_mnl-exp_ as the gold standard, VTI_auto_ represented an 11.4% underestimate and VTI_mnl-train_ a 7.8% underestimate of true VTI. All D_LVOT_ measurements were done by trainees as no automatic measurement was possible. This measurement demonstrated a wide range of values (1.9–2.9 cm) with ± 20.8% variability. By contrast, D_LVOT_ by both expert echocardiographers was only 2.2 cm.

### Precision and accuracy of CO measurement

While CO_auto_ and CO_mnl-train_ correlated well (*ρ* = 0.76 p < 0.001), Fig. 2c, both mean CO_auto_ (4.5 ± 1.2 L/min) and mean CO_mnl-train_ (4.6 ± 1.0 L/min) underestimated mean CO_mnl-exp_ (5.7 ± 2.5 L/min). Bland–Altman matched-pairs analysis showed no significant difference between the paired means (mean difference = 0.1, *p* = 0.45), indicating no systematic bias between the two methods (Fig. 2d). Additionally, 95% of the paired differences were expected to fall between − 1.54 and + 1.86. Interestingly, the measurement range across raters was wider with CO_auto_ (4.2 vs. 3.5 L/min) while inter-rater coefficient of variation was also higher with automatic measurement (26.7% vs. 21.7%).

### Sensitivity analysis for VTI inter-rater reproducibility

**Fig. 3:**
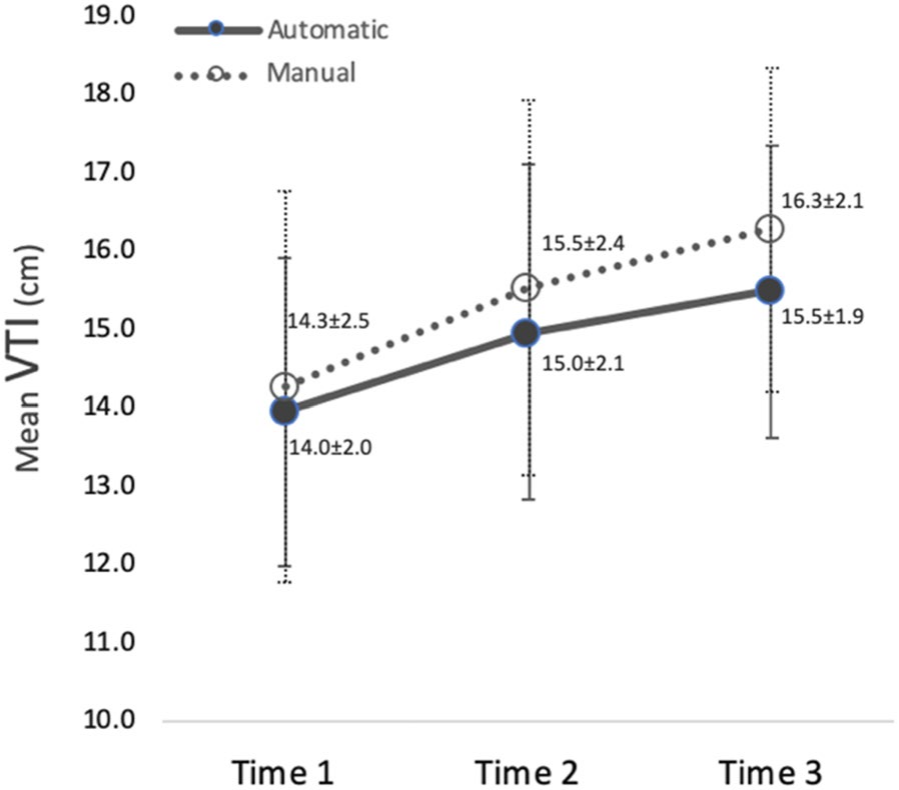
Physiologic changes in VTI during testing represented as mean measurements of both automatic and manual methods in three consecutive time intervals

To evaluate the effects of physiologic variability on VTI precision and reproducibility, we analyzed the physiologic alterations of VTI during the 3 h of our study by splitting participants into three groups that would take measurements at each hour and compared their VTI means to each other. We then evaluated reproducibility within each time interval for comparison to the previous time interval’s estimates. The VTI means across the three time intervals were 14.3 cm, 15.5 cm, and 16.3 cm for manual measurements and 14.0 cm, 15.0 cm, and 15.5 cm for automated measurements. These differences in means were not statistically significant. The inter-rater standard deviations (SDs) within the three time intervals were 2.49, 2.40 and 2.06 cm for manual measurements and were 1.96, 2.14, and 1.86 cm for automated measurements. The corresponding coefficient of variation (CV) estimates were 17.4%, 15.5%, and 12.7% for manual measurements and were 14.0%, 14.3%, and 12.0% for automated measurements (Fig. 3). The pooled SD combining the three time intervals was 2.31 cm with a CV (pooled SD divided by overall mean) of 15.0% for manual measurements. Meanwhile, pooled SD was 1.98 cm with a CV of 13.4% for automated measurements across all three time intervals. These estimates were close to previous estimates that ignored potential time effects.

## Discussion

In this study, we evaluated the ability of novice critical care trainees to quantify CO using both manual and automated VTI measurements. We then compared their measurements to those attained by our control group of expert echocardiographers. We found that the feasibility of using AI-augmented image acquisition was excellent and, as defined by the coefficient of variation, the precision of VTI capture was higher with automatic VTI tracing when compared to manual VTI tracing.

Our relatively small, prospective study addresses several important issues related to the use of POCUS in cardiac output assessment. First, our study demonstrates that the ability to obtain relatively adequate measurements required for CO estimation does not require absolute expertise but a short, structured course with opportunities for hands-on practice. All 28 trainees were able to obtain adequate PLAX and AP5c views, resulting in 100% feasibility for image acquisition despite minimal US training. However, our reported feasibility for VTI is significantly higher than those previously published [10–12] even when accounting for training level [10]. This may be because all examinations were obtained on a single healthy volunteer who was hemodynamically stable and adequately positioned by experienced ultrasonographers, thus facilitating image acquisition. However, it must be noted that medical professionals with minimal training can obtain adequate parasternal and apical views 88% of the time when assessing critically ill patients [13]. These data further solidify the need for including POCUS education in medical training [14, 15].

Our study also considered accuracy and precision separately when measuring cardiac output and VTI because their distinction is clinically relevant. For example, accuracy of cardiac output has diagnostic implications in determining etiologies for specific hemodynamic states such as cardiogenic or distributive shock. By contrast, precision of cardiac measurements carries therapeutic implications. As an example, knowing with statistical confidence that SV or CO changed in response to an intervention, independent of SV or CO accuracy, is crucial for determining treatment effectiveness. In our study, trainees had larger measurement variations for D_LVOT_ (1.9 to 2.9 cm) when compared to expert echocardiographers. Interestingly, both VTI_mnl-train_ and VTI_auto_ underestimated true VTI as determined by expert echocardiographers. As a result, both manual and automatic cardiac output measurements had poor accuracy compared to the standardized control. Measurement variation likely has multiple sources, including human factors [16, 17], ultrasound factors [18], and intrinsic fluctuation secondary to patient physiology (e.g., respiratory variation). However, ultrasound and patient factors were less likely to contribute to measurement variability in our study since trainees and expert echocardiographers used the same machines and obtained measurements from only one patient. Therefore, differences in the coefficient of variation are likely to be operator dependent. Other differences noted in our study include the number of beats sampled before and after an intervention to detect a 10% SV change with statistical confidence. Expert echocardiographers, automatic VTI tracing, or manual VTI tracing by trainees were 10, 27, and 34, respectively [19]. These beat numbers are greater than the recommended 3 in clinical practice [20].

Hemodynamic assessment in our study was based on velocity time integral, which has been criticized for its difficulty of attainment in critical care settings. For example, the reported feasibility of VTI in the intensive care unit (ICU) ranges from 37 to 90% [10, 11, 21], likely due to inter-rater variability [10]. Other downsides to using VTI to measure CO include its dependence on insonation angles, which presents their own set of challenges [22–24]. Nevertheless, VTI is commonly used in tracking changes in SV and CO in critically ill patients [22, 25, 26] and has also demonstrated the ability to predict outcomes in select populations [27]. Together with other Doppler-based measures [28], VTI has become an increasingly reliable metric to assess fluid responsiveness in shock [29, 30]. Indeed, our results showcase VTI’s reproducibility between trainees and expert operators. However, it should be noted that VTI_auto_ was more reproducible across raters than VTI_mnl-train_, demonstrating narrower measurement range, smaller standard deviation, and lower coefficient of variation. Taken together, these results show that automation could further solidify VTI as a reliable metric for cardiac output measurements and reduce operator-dependent variability in clinical practice.

AI-assisted programs not only augment user experience, but also redefine the capabilities of US in a critical care setting [31]. As an example, automated VTI measuring systems highlight the importance of real-time feedback by aiding fatigued users, who may be more prone to making errors, perform routine echocardiographic tasks such as spectral envelope tracing, chamber volume estimations, and tracing endomyocardial boundaries. Equally important, AI can help determine view quality and assist directly with US image acquisition by guiding users towards optimal viewing angles. In fact, Zhang et al. trained an algorithm through a convolutional neural network model to accurately identify 23 viewpoints and segment cardiac chambers across 5 different views. The model was able to perform these tasks correctly 96% of the time. Even more impressive was its ability to flag views with partially obscured cardiac chambers [32]. As demonstrated in our study, our US device was able to identify foreshortened, or otherwise inadequate, 5-chamber views with poor aortic outflow jets and guide the user to an acceptable view through a series of coded Doppler box colors. This live feedback can provide users with accurate measurements for clinical decision-making in addition to training users to improve their US skills through a positive feedback loop. Therefore, we believe that educating operators along with the development of AI will not only train operators to use ultrasound, but also train ultrasound itself to tolerate different operators.

There are numerous studies demonstrating the utility of AI in estimating left ventricular hypertrophy, spectral wave tracing, right and left ventricular ejection fraction, three-dimensional chamber volume analysis, and regional wall motion [33–41]. However, LVOT VTI automation remains understudied. To our knowledge, there is only one report assessing automated VTI (VTI_auto_) accuracy, which used an animal model for hemorrhagic shock [42]. In this study, however, feasibility was low (60%) and the correlation coefficient between VTI_auto_ and PAC thermodilution was moderate at best (*r* = 0.66). As a result, our study will add to the somewhat sparse body of knowledge surrounding automated VTI and hopefully pave a path for larger, structured prospective studies that can further evaluate AI approaches in POCUS for hemodynamic assessments.

There are several limitations to our study. First, manual and automated measurements of CO are highly dependent upon D_LVOT_, so any inaccuracies in D_LVOT_ are amplified by the modified Bernoulli equation. This was a large source of inaccuracy in CO measurement by the trainee group and could be improved by incorporating D_LVOT_ measurement into the automation model. All exams were also done on one standardized volunteer to standardize comparison between participants and experts. While we minimized movement and stress on our study subject, stroke volume, and therefore VTI, can change during the study period, warranting the need for more frequent control measurements by expert echocardiographers. Additionally, our choice to only study novice sonographers inherently decrease endpoint measurement accuracy when compared to experienced echocardiographers. However, we chose this population for the study because there is no standardized US training in the ICU, and we sought to shed light on this subgroup of clinicians. In addition, novice US users are likely to benefit the most from AI assistance since they lack knowledge on US standard operating procedures. We also did not assess how long it took trainees to obtain appropriate LVOT VTI images as it was beyond the scope of our study. However, previous studies with participants of similar proficiency with US found the median acquisition time to be about 2 min [18, 19]. Further studies are needed to assess whether automated processes can help hasten image acquisition time. Finally, our study is small, with only 28 participants assessing VTI on one study patient who was healthy and hemodynamically stable. Therefore, future studies are needed to assess whether AI-assisted POCUS is a viable option in a clinical setting with patients with varying pathologies or experiencing active hemodynamic instability. Nevertheless, the variability in VTI measurement that we encountered between novice and expert sonographers remained significant despite a small sample size. Further evaluation of AI-augmented VTI to improve accessibility, reliability, and accuracy will be needed to fully appreciate the effects of AI on POCUS in critical care.

## Conclusion

The role of Doppler-based evaluation of cardiac output has not been firmly established in daily practice with POCUS, primarily due to difficulty with acquiring adequate images, concern about the ability to precisely measure VTI, and the complexities associated with calculating stroke volume. Automation may offer new, efficient ways to assess CO by addressing each of these aforementioned challenges and allow for more reliable and simpler repetitive hemodynamic assessment that can be performed by an operator with limited ultrasonographic experience. In our study, automatic VTI tracing improved the coefficient of variation in novice critical care trainees compared to manual tracings. These results support the use of AI-augmented image acquisition and may have implications for evaluating the hemodynamic interventions using VTI and SV.

## Data Availability

The datasets used and/or analyzed during the current study are available from the corresponding author on reasonable request.

## Abbreviations

AI: Artificial intelligence
AP5c: Apical 5-chamber
CO: Cardiac output
CV: Coefficient of variation
D_LVOT_: Left ventricular outflow tract diameter
ESICM: European Society of Intensive Care Medicine
HR: Heart rate
ICU: Intensive care unit
LVOT: Left ventricular outflow tract
PAC: Pulmonary artery catheter
PLAX: Parasternal long axis
POCUS: Point-of-care ultrasound
SD: Standard deviation
SV: Stroke volume
UCLA: University of California, Los Angeles
US: Ultrasound
VTI: Velocity time integral
